# Exploring the Potential of MBenes Supercapacitors: Fluorine-Free Synthesized MoAl_1–*x*_B with Ultrahigh Conductivity and Open Space

**DOI:** 10.1021/acsami.3c04301

**Published:** 2023-07-05

**Authors:** Shudan Wei, Xiaojun Lai, Girish M. Kale

**Affiliations:** School of Chemical and Process Engineering, University of Leeds, LS2 9JT Leeds, United Kingdom

**Keywords:** MBenes, supercapacitor, fluoride-free, etching, high conductivity, layered structure

## Abstract

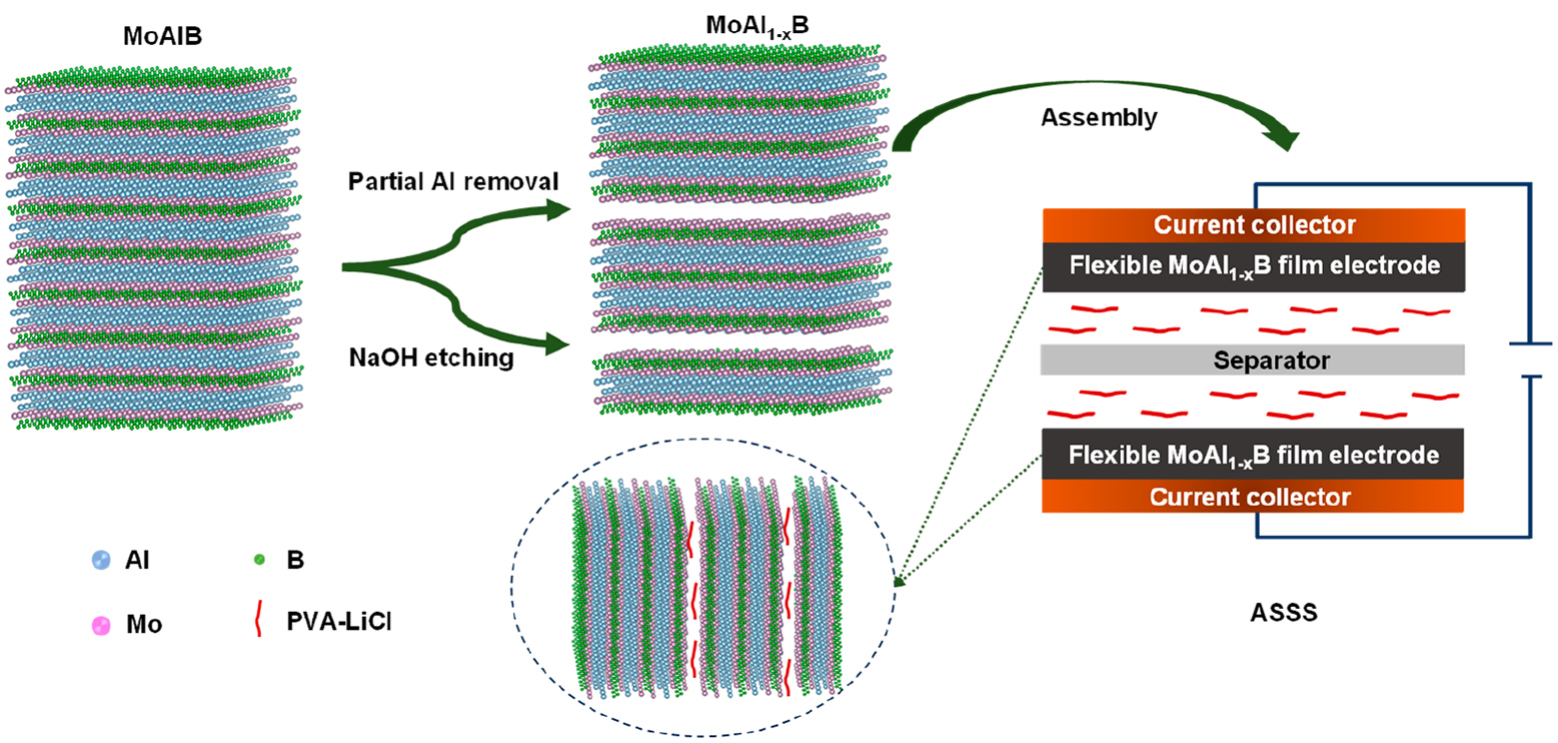

The present study describes the synthesis of multilayered MBenes MoAl_1–*x*_B with different degrees of Al deintercalation using a mild, fluorine-free approach of dilute alkali to remove Al from MoAlB. We propose an etching route and compare it to conventional fluoride etching products. Additionally, the study explores the possible application and energy storage mechanism of MBenes in supercapacitors, marking the first investigation of its kind. At room temperature, 1/24-MoAl_1–*x*_B with terminal groups −OH exhibits ∼25% Al removal in 1 wt % NaOH for 24 h, outperforming traditional etching technology. Increasing the Al removal exposed more open space, resulting in a higher capacitance. Compared to LiF/HCl-MoAl_1–*x*_B (etched by LiF + HCl), 1/24-MoAl_1–*x*_B has a higher energy storage capability. The multilayered 1/24-MoAl_1–*x*_B film electrode exhibits ultrahigh conductivity with a rapid relaxation time of 0.97 s and high areal capacitance (2006.60 mF cm^–2^) while maintaining 80.2% capacitance after 5000 cycles. The MoAl_1–*x*_B all-solid-state supercapacitor (ASSS) delivers a high capacitance of 741.6 mF cm^–2^ at 1 mV s^–1^ for a single electrode and exhibits stable capacitance even at a 90° bending angle, highlighting its potential practical use. Our research represents an important step in the synthesis of MBenes and highlights their potential applications in supercapacitors.

## Introduction

Supercapacitors have gained significant attention owing to their superior high-power density and long cycle life when compared with other energy storage devices such as batteries and fuel cells.^1,2^ These devices can be categorized into electric double-layered capacitors (EDLCs) and pseudocapacitors (PCs) based on the charge storage mechanism of the electrode materials during an electrochemical process.^3^ While carbon-based EDLC materials demonstrate good cycling stability, they have low specific capacity.^4^ On the other hand, pseudocapacitive electrode materials such as oxides can overcome this limitation, but they suffer from rapid capacity fading with repeated charge–discharge cycles.^5^ To address this challenge, researchers have explored metal sulfides, nitrides, carbides, and boride-based electrode materials due to their improved electronic conductivity for various electrochemical applications.^6^

By applying joint density functional theory (JDFT) to the electrochemical system, Zhan et al.^7^ found that 2D-boron sheets can exhibit a specific capacitance of 400 F g^–1^. They concluded that boron-based nanosheets perform better than graphene for supercapacitors due to their low molecular weight and metallic nature. Furthermore, boron-doped nanosheets have been of great interest in energy storage applications, as Chi et al.^8^ have reported boron-doped graphene for high-voltage aqueous supercapacitors. The high specific capacitance of 336 F g^–1^ was found for boron-doped mesoporous graphene where it reduced to 169 F g^–1^ at a current density of 0.1 A g when boron was removed by calcination.^9^ This study revealed that boron plays a vital role in the charge storage capability. In this aspect, most recently, 2D-layered boron-based materials have been widely researched for energy storage applications.

The development of energy storage device is dependent on the design of novel electrode materials.^10^ A new group of transition metal boride layered nanomaterials (MBenes) is explored highly due to their exceptional electronic conductivity, outstanding stability, and rich surface chemistry.^11,12^ 2D multilayer MBenes are derived by chemically etching and exfoliating 3D MAB phases, which typically contain a transition metal (M), IIIA and IVA group elements (A), and boron (B). The first report on MBenes dates back to 2017,^13^ where they were considered analogous to early transition metal carbides, nitrides, and carbonitrides (MXenes). On the other hand, MBenes have received less attention than MXenes but have immense potential in many areas which benefit from the multiple valence state of the boron atom and its electron-deficient nature.^14^ Theoretical calculations and preliminary experiments suggest a plethora of applications for 2D MBenes in magnetic devices,^15^ electrocatalysis,^11,16^ and batteries,^13,17,18^ making the exploration of these classes of compounds promising. MBenes are also predicted to build newer, more efficient supercapacitors with long-cycle stability and high-power density.^14^ MAB, specifically MoAlB, exhibit extremely low electronic resistance,^19^ but their use, particularly in the bulk form, is often hindered by poor exposure to active sites due to their low specific surface area. Numerous attempts have been made to synthesize its deintercalation products (MBenes) by etching aluminum layers from the MoAlB phase. Several studies have demonstrated that the etching process can effectively remove approximately 25% of the aluminum content in MoAlB,^20−22^ leading to improved electrochemical properties. However, current etching methods usually involve the use of fluoride etchants or high corrosion solutions at elevated temperatures,^19,23^ which are either not cost-effective or environmentally unfriendly and pose safety hazards. As a result, the development of a more cost-effective and environmentally friendly etching process remains an open challenge. One potential approach to improving the electrochemical properties of MoAlB is by deintercalation of aluminum from MoAlB with larger spacing. However, to the best of our knowledge, the promising application and energy storage mechanism of MBenes in supercapacitors are yet to be reported.

**Figure 1:**
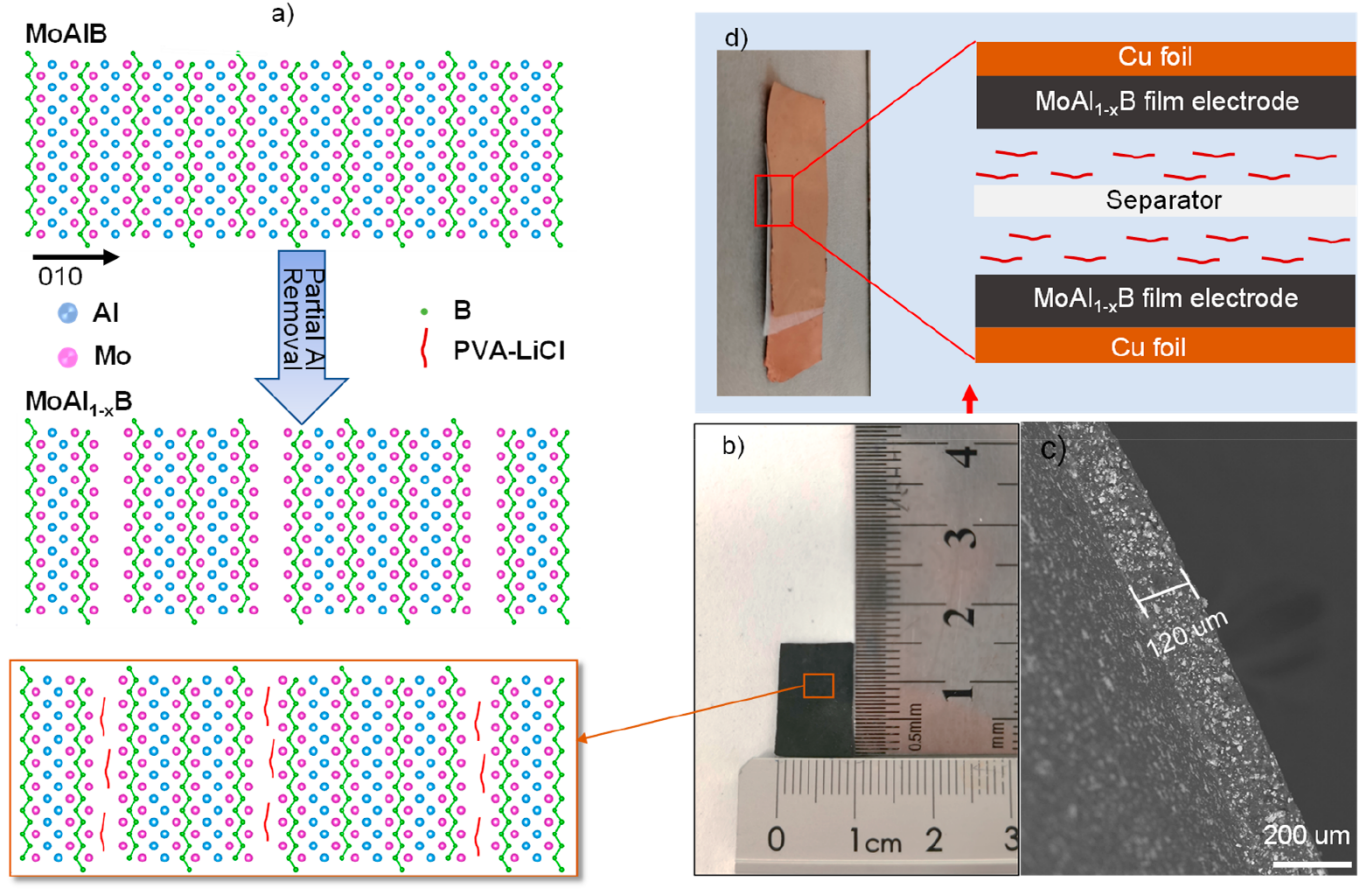
(a) Schematic synthesis process for MoAl_1–*x*_B, (b) photograph of MoAl_1–*x*_B film electrode (PVA–LiCl is the solid-state poly(vinyl alcohol)–LiCl electrolyte), (c) SEM image of electrode thickness, and (d) assembly of ASSS.

In this study, multilayered MBenes MoAl_1–*x*_B with different Al deintercalation rates (up to ∼25%) were synthesized by removing Al from MoAlB using dilute alkali NaOH and traditional fluoride etchant LiF/HCl (Figure 1). The etching route and chemical reactions were investigated, and the findings indicate that 1/24-MoAl_1–*x*_B with −OH terminal groups exhibited approximately 25% removal of Al at room temperature for 24 h using a 1 wt % NaOH etching solution, outperforming traditional etching solutions.^20−22^ Meanwhile, LiF/HCl-MoAl_1–*x*_B (etching with LiF/HCl) displayed a lower rate of Al removal compared to 1/24-MoAl_1–*x*_B. Following on, the properties and energy storage mechanism of MBenes in supercapacitors were investigated for the first time. The capacitance of MoAl_1–*x*_B enhanced with the increase of the Al defective rate due to the open space. The 1/24-MoAl_1–*x*_B film electrode exhibited ultrahigh conductivity, superior cycle performance, and high areal capacitance (2006.60 mF cm^–2^). 1/24-MoAl_1–*x*_B had a higher and more stable energy storage capability than LiF/HCl-MoAl_1–*x*_B. Diffusion-controlled and surface-controlled mechanisms coexisted during charge–discharge. Additionally, an all-solid-state supercapacitor device (Figure 1b–d) based on the flexible 1/24-MoAl_1–*x*_B film electrode delivered significant areal capacitance, high energy density, and power density. This research introduces the gentlest and fluoride-free methods for synthesizing MBenes-based materials, highlighting their significant potential for utilization in supercapacitors.

## Experimental Section

### Preparation of MoAl_1–*x*_B

Different Al removal rates in multilayer MoAl_1–*x*_B have been achieved by immersing 200 mg of commercial MoAlB (200 mesh, Laizhou Kai Kai Ceramic Materials Co., Ltd.) into 20 mL of 1 wt % NaOH at room temperature (RT) and stirred for 1, 12, and 24 h, 10 wt % NaOH for 24 h, and 30 wt % NaOH at 50 °C for 48 h. The products were denoted as 1/1-MoAl_1–*x*_B, 1/12- MoAl_1–*x*_B, 1/24-MoAl_1–*x*_B, 10/24-MoAl_1–*x*_B, and 30/48/50-MoAl_1–*x*_B, respectively. LiF/HCl-MoAl_1–*x*_B was obtained by etching MoAlB in 6 M HCl + 1.2 g of LiF usually used for preparing MXenes at 35 °C for 24 h.^24^ The prepared materials were rinsed with deionized water to pH ∼ 7, then separated from the solution via centrifugation, and dried at 60 °C overnight.

### Characterization

The elemental composition and morphological analysis were characterized for the prepared sample by a scanning electron microscope (SEM, Hitachi SU8230) equipped with energy-dispersive X-ray analysis (EDX). The X-ray diffraction (XRD, Bruker D8) has been performed to confirm the formation of the prepared phase and determine its crystallinity. X-ray photoelectron spectroscopy (XPS, SPECS EnviroESCA) has been employed to estimate the surface composition and electronic state of the elements in the prepared sample. Atomic absorption spectroscopy (AAS, Agilent FS 240) was used to further confirm the Al etching rates, and the specific surface area of samples was measured by a surface area and porosity analyzer (Micromeritics TriStar 3000). Surface termination groups have been verified by a Fourier transform infrared spectrometer (FTIR, Thermo Scientific Nicolet iS10). The electrochemical characterization and data analysis were performed using electrochemical workstations (Solartron SI 1280 and SI 1260) equipped with software Corrware and Zplot (Scribner Associates Inc.).

### Preparation of MoAl_1–*x*_B Film Electrodes

The thick film electrodes were prepared by a mechanical process of a premixed slurry, containing MoAl_1–**x**_B/MoAlB powder, conductive agent acetylene black (AC), and PTFE binder (60 wt % in H_2_O). The slurry contained 70 wt % MoAl_1–*x*_B, 10 wt % PTFE, and 20 wt % AC. The slurry was rolled into a film with a thickness of about 120 μm and then tailored into rectangular sheets (1 × 1.5 cm^2^). These electrodes were dried at 60 °C for 24 h. The mass density for the active materials per unit area was approximately 30 mg cm^–2^.

### Fabrication of All-Solid-State Supercapacitor (ASSS)

The ASSS was fabricated by sandwiching two pieces of rectangular 1/24-MoAl_1–*x*_B film electrodes with solid-state poly(vinyl alcohol) (PVA)–LiCl electrolyte and separated by an NKK membrane (PP, MPF). Specifically, 1 g of LiCl and 0.5 g of PVA powder were added to 5 mL of deionized water. Then, the mixture was heated at 70 °C under stirring until it became a transparent gel. The gel was cooled naturally to room temperature and cast onto one side of the 1/24-MoAl_1–*x*_B film electrode. After solidification, two copper foils were attached as current collectors and directly connected to the electrochemical station.

### Electrochemical Measurements

Electrochemical experiments of film electrodes were performed in a three-electrode setup and a two-electrode setup. As for the three-electrode setup, the synthesized film electrode is considered as the working electrode, 1 × 1.5 cm^2^ Pt mesh as the counter electrode, 1 M Na_2_SO_4_/1 M H_2_SO_4_/2 M NaOH as electrolyte, and Ag/AgCl (saturated KCl) as a reference electrode. A separator was sandwiched between a couple of film electrodes in a two-electrode setup. All electrochemical measurements for ASSS were performed in a symmetric two-electrode configuration. The electrochemical studies for supercapacitor application were done using cyclic voltammetry (CV), galvanostatic charge–discharge (GCD), and electrochemical impedance spectroscopy (EIS). CV curves were obtained at different scan rates: 1, 2, 5, 10, and 20 mV s^–1^ in a suitable voltage window. EIS was demonstrated in the Nyquist plot in the frequency range from 0.1 to 10^6^ Hz by applying a fixed potential of 20 mV. GCD curves were obtained at different current densities ranging from 2 to 24 mA cm^–2^. The capacitance, energy density, and power density of the film electrodes and the devices were calculated by eqs S1–S10 of the Supporting Information. The flexible properties of ASSS have also been tested by comparing the CV curves after bending the flexible device from 0° to 30°, 60°, and 90°.

## Results and Discussion

### Preparation and Characterization

**Figure 2:**
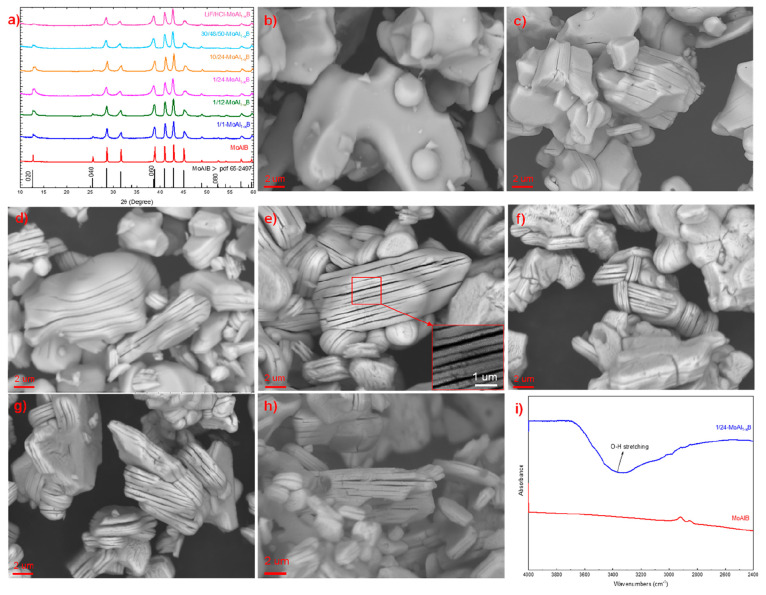
(a) XRD patterns for MoAlB before and after etching. SEM photographs of (b) MoAlB, (c) 1/1-MoAl_1–*x*_B, (d) 1/12-MoAl_1–*x*_B, (e) 1/24-MoAl_1–*x*_B (inset is the enlarged SEM image), (f) 10/24-MoAl_1–*x*_B, (g) 30/48/50-MoAl_1–*x*_B, and (h) LiF/HCl-MoAl_1–*x*_B. (i) FTIR spectra of pristine MoAlB and 1/24-MoAl_1–*x*_B.

**Figure 3:**
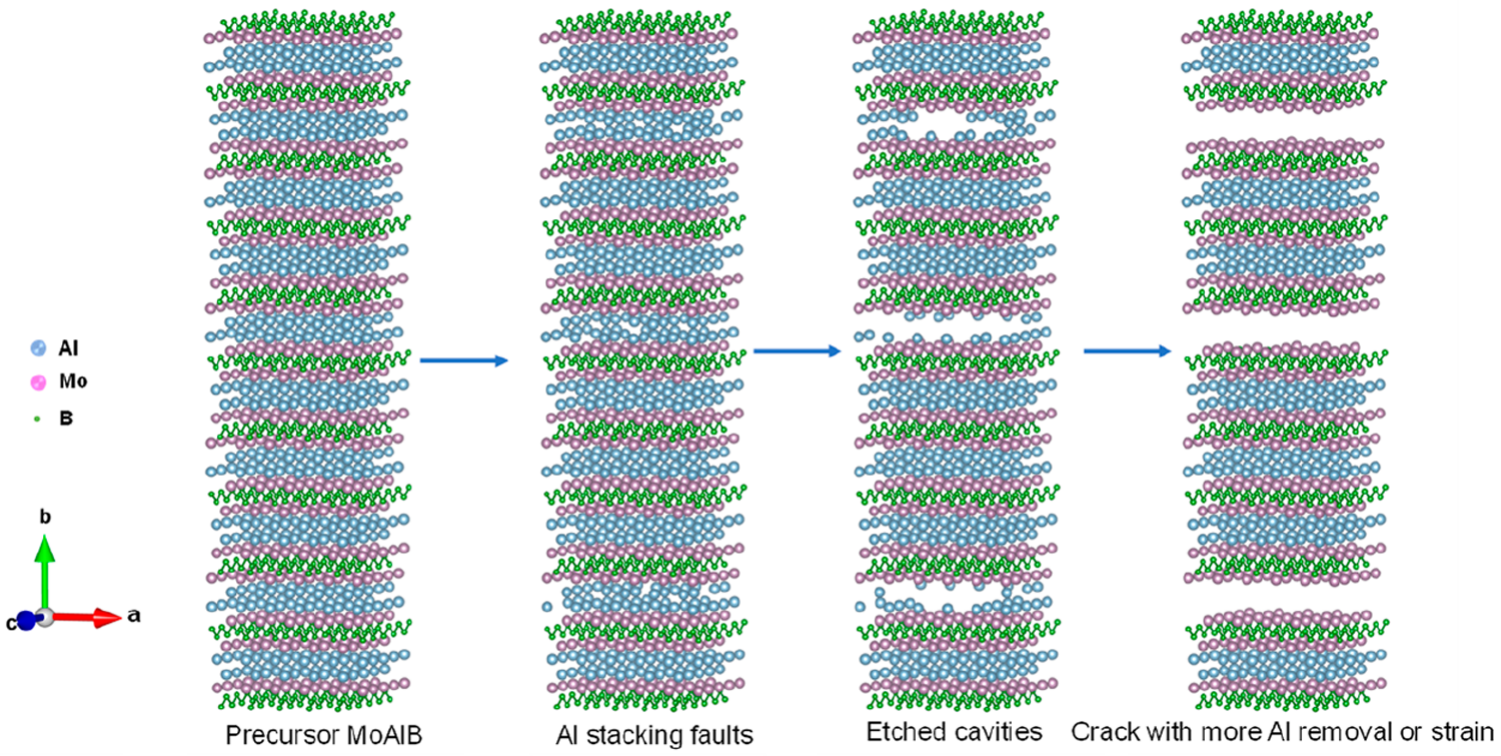
Schematic representation of the aluminum removal process from MoAlB.

Highly crystalline precursor MoAlB particles were confirmed by sharp diffraction peaks in their XRD pattern (Figure 2a), and solid particle morphology without layers was validated by SEM images (Figures 2b and S1). SEM-EDX analysis revealed a 0.992:1 Al:Mo ratio (Table 1). Etching in 1 wt % NaOH caused a decrease in the Al:Mo ratio from 0.992:1 to 0.752:1. AAS also confirms an ∼25% Al removal after 24 h, indicating partial etching of the Al layer of MoAlB. XRD analysis showed its crystal structure is retained; however, the (0*k*0) reflections weakened with increasing etching time. The (040) reflection also weakened and almost disappeared after 24 h of etching, and higher temperature or longer etching time did not further improve the etching efficiency, indicating that the Al removal rate reached equilibrium. Despite previous reports showing only ∼25% etching by fluoride treatment or concentrated alkaline,^20,25^ our study found that using 1 wt % NaOH at room temperature was the mildest method that also achieved ∼25% Al removal. SEM images in Figure 2c–e show looser structures formed with increasing etching time, but the slab thickness remained stable from 1 to 24 h. After etching for 24 h, MoAl_1–*x*_B slabs with thicknesses ranging from 50 to 500 nm exhibited periodic gaps with 10–200 nm spacings. We propose an etching pathway (Figure 3) based on the retained XRD patterns and SEM results, where Al deintercalation from some layers causes stacking faults to form in MoAlB. As more Al is removed, stacking fault density increases in the same Al-defective layers due to the higher surface free energy than other intact Al-occupied layers, resulting in crack formation in a multilayer structure. The Al layers here were removed in a staged manner, employing a similar route as the previous report for MoAlB etching by concentrated alkali.^20^ The remaining MoAl_1–*x*_B slab can be viewed as (MoB)_2_-(Al_2_(MoB)_2_)_*n*_-Al_2_(MoB)_2_ (Figure 3). Bubbles formed during etching reveal the overall reaction for the removal of Al from MoAlB (eq 1):
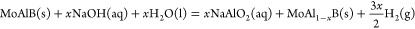
1Higher concentrations of NaOH and temperature did not increase the gap size or get thicker slabs (Figure 2f,g), so we introduced fluoride (LiF + HCl) to in situ generate HF to further study etching chemistry. XRD patterns and SEM images (Figure 2h) showed no significant differences, and SEM-EDX results indicated a lower Al removal. Treatment with LiF + HCl revealed evidence of etching, but some dark phases appeared (Figure S1), which increased Al retention in MoAl_1–*x*_B and stopped further etching due to surface coatings of AlF_3_ and Al oxide.^25^ The dark phase EDS spectrum showed a high Al and F atomic ratio (Figure S1 and Table S1), indicating Al removal relied on the reaction of HF and Al atoms, generating insoluble AlF_3_ after etching (eq 2).^26^
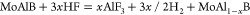
21 wt % NaOH solution is the preferred etchant for MoAlB due to its cost-effectiveness and mildness compared to previous etchants for the MXene phase^27^ and MAB phase etchants.^12^ According to density functional theory (DFT) computer results, the energy required to generate the first Al defect in the Al layer of MoAlB is 5.03 eV, which is lower than that of the Ti_3_AlC_2_ case (6.38 eV).^22,28^ Therefore, it is more feasible to remove Al atoms from MoAlB. The use of 1 wt % NaOH to etch Al from MoAlB is a crucial step toward achieving chemical delamination of a MAB phase^12,20−22^ and even a MAX phase,^27,29−31^ and it will broaden their usage in various research fields such as electrocatalysis, energy storage, and biochemistry.^23^ After more aluminum was removed, we observed an increase in the specific surface area of MoAl_1–*x*_B, as shown in Table 1. This enhancement promotes improved ion diffusion and contributes to better supercapacitor performance. Among the etching methods, LiF/HCl etching resulted in the highest Langmuir surface area due to the presence of smaller particles of AlF_3_ and aluminum oxide. However, the aluminum removal rate of LiF/HCl-MoAl_1–*x*_B was found to be lower compared with the products obtained from NaOH etching. Furthermore, the presence of the terminal functional group −OH on the surface of MoAlB after etching, as shown by the band at ∼3350 cm^–1^ of FTIR spectra (Figure 2i), is similar to what has been observed on the surface of MXene slabs when selectively etching the A element from MAX phases.^29−31^

**Table 1:** Atomic Ratio for Al:Mo (EDX), Al Dissolution Rate (AAS), and Langmuir Surface Area of Different Samples

samples	MoAlB	1/1/-MoAl_1–*x*_B	1/12-MoAl_1–*x*_B	1/24-MoAl_1–*x*_B	10/24-MoAl_1–*x*_B	30/48/50-MoAl_1–*x*_B	LiF/HCl-MoAl_1–*x*_B
Al:Mo (EDX)	0.992:1	0.908:1	0.824:1	0.752:1	0.758:1	0.747:1	0.778:1
Al dissolution rate (AAS, %)	0	10.4	18.7	25.3	24.7	25.8	22.5
Langmuir surface area (m^2^ g^−1^)	0.3446	0.4450	0.6363	0.6968	0.6834	0.6990	0.7244

**Figure 4:**
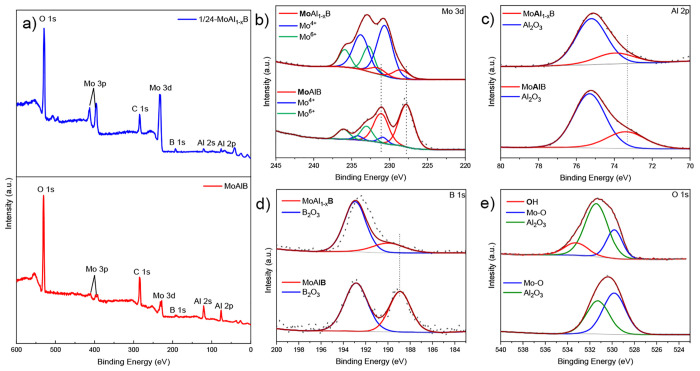
(a) XPS spectra and high-resolution spectra with peak fitting of (b) Mo 3d, (c) Al 2p, (d) B 1s, and (e) O 1s regions for MoAlB before (lower spectra) and after (upper spectra) etching in 1 wt % NaOH for 24 h (1/24-MoAl_1–*x*_B).

The prevalence of the species concerned is further corroborated by X-ray photoelectron spectroscopy (XPS) analysis, presented in Figure 4 and Tables S2–S5. After etching for 24 h, the Al:Mo ratio measured by XPS was down to 0.764:1 in 1/24-MoAl_1–*x*_B (∼5% error in the quantitative analysis of XPS). The fitted peaks at a binding energy (BE) of 228.53 eV (231.58 eV) are assigned to MoAl_1–*x*_B (Figure 4b) and are shifted by 0.75 eV higher than that for the precursor MoAlB.^32^ A similar shift has also been observed for the M (metal) element when selectively etching the A element in MAX phases, forming MXenes.^33^ As shown in Figures 4c and 4d, the peaks of Al 2p and B 1s for MoAl_1–*x*_B are also shifted to higher values due to the introduction of the electron-withdrawing group −OH and the increasing valence of the remaining Al. The fitted XPS spectra of the O 1s regions also show the presence of the terminal group −OH after etching (Figure 4e), which is consistent with the FTIR results.

### Electrochemical Properties of MoAl_1–*x*_B

**Figure 5:**
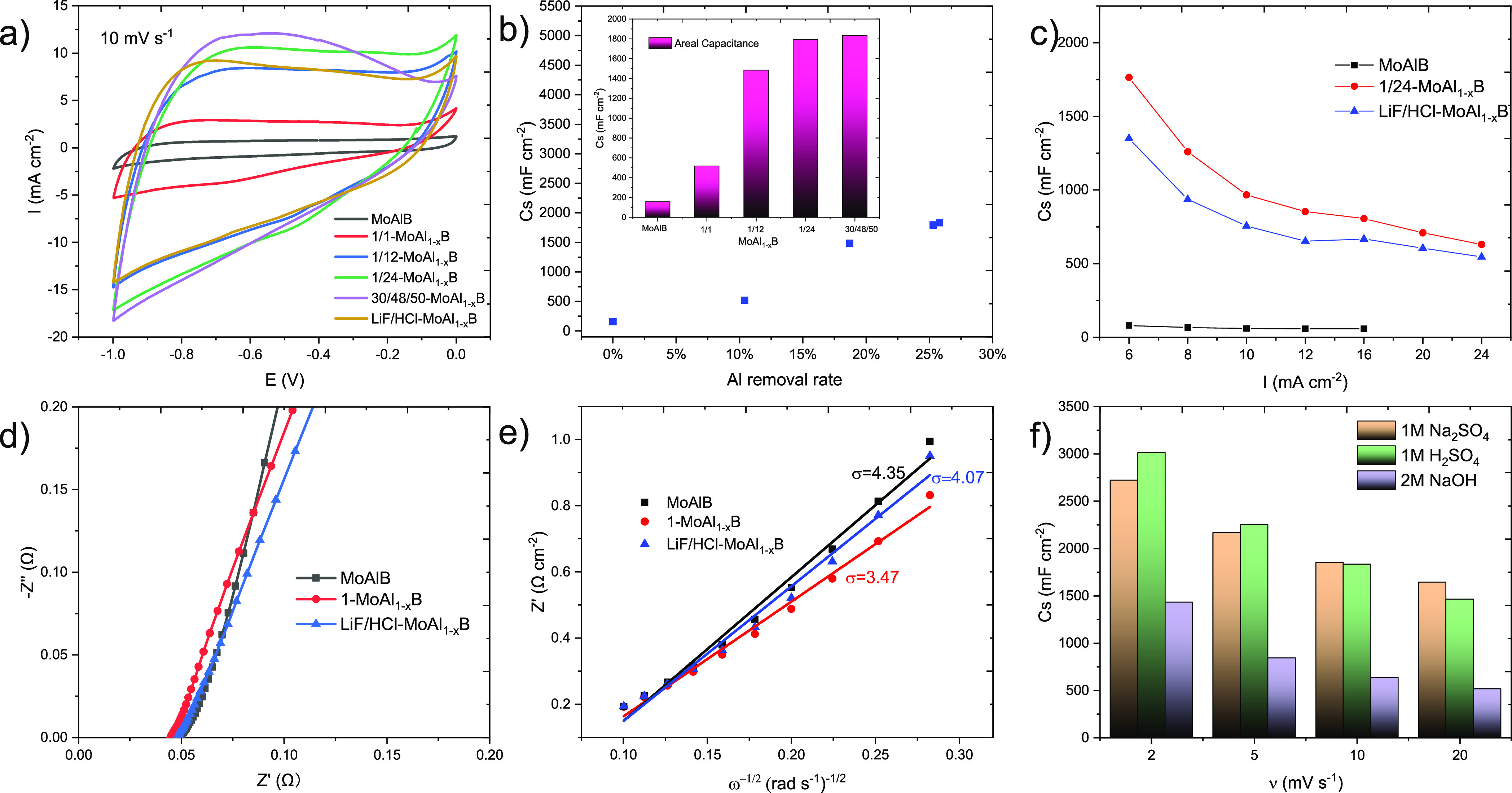
Electrochemical measurements in 3-electrode setup: (a) CV curves for MoAlB and MoAl_1–*x*_B at scan rate 10 mV s^–1^ in 1 M Na_2_SO_4_; (b) variation of areal capacitance of MoAl_1–*x*_B with Al removal rate (10 mV s^–1^, NaOH etching only), insert is the detailed capacitance values; (c) areal capacitance for MoAlB, 1/24-MoAl_1–*x*_B, and LiF/HCl-MoAl_1–*x*_B at different charge–discharge speeds; (d) Nyquist plots for MoAlB, 1/24-MoAl_1–*x*_B, and LiF/HCl-MoAl_1–*x*_B; (e) the fitted lines between *Z*_re_ and ω^–1/2^ for MoAlB, 1/24-MoAl_1–*x*_B, and LiF/HCl-MoAl_1–*x*_B; (f) areal capacitance of 1/24-MoAl_1–*x*_B in 1 M Na_2_SO_4,_ 1 M H_2_SO_4_, and 2 M NaOH.

The potential of MBenes MoAl_1–*x*_B for supercapacitor applications was initially evaluated by investigating the effect of different Al etching rates on the properties of MoAlB and the fluoride etching product in a three-electrode system. Figures 5a and 5b demonstrate that the areal capacitance of MoAl_1–*x*_B at a scan rate of 10 mV s^–1^ increases as the rate of Al removal increases. However, the capacitance of 1/24-MoAl_1–*x*_B and 30/48/50-MoAl_1–*x*_B remains almost the same due to a very similar Al removal rate (∼25%). Figures 5c and S2 demonstrate that MoAl_1–*x*_B exhibits significantly improved performance compared to its precursor MoAlB due to a larger surface area, better exposure of active sites after etching, and more space available for electrolyte infiltration. Furthermore, 1/24-MoAl_1–*x*_B outperforms LiF/HCl-MoAl_1–*x*_B due to higher Al removal and lower conductivity of AlF_3_ remaining in LiF/HCl-MoAl_1–*x*_B. Moreover, the presence of the terminating −OH in 1/24-MoAl_1–*x*_B enhances its supercapacitor properties. Bulk MoAlB exhibits lower resistivity than MoB (0.36–0.49 μΩ·m^19^ vs ∼6 μΩ·m^34^). As metal Al acts as “electron bridges” in MoAlB bulk materials, the reserved Al ensures high interlayer conductivity after partial Al removal from MoAlB. The solution resistance of MoAlB and MoAl_1–*x*_B is extremely low, as shown in Figure 5d. Analyzing the diffusion coefficient (*D*) by fitting the real part of the impedance (*Z*_re_) with the square root of the radial frequency (ω^–1/2^) according to eqs S11 and S12(35) based on the electrochemical impedance spectroscopy (EIS) results, it can be concluded that 1/24-MoAl_1–*x*_B exhibits the higher diffusion coefficient *D* and significantly enhanced ion transport compared to MoAlB and LiF/HCl-MoAl_1–*x*_B (Figure 5e), indicating higher diffusion coefficient *D* and enhanced ion transport after more Al etching, making it the best option for further study of MBenes supercapacitors.

The electrochemical performances of the as-prepared 1/24-MoAl_1–*x*_B film electrodes were evaluated by using 1 M Na_2_SO_4_, 1 M H_2_SO_4_, and 2 M NaOH as an electrolyte. The suitable working window in 1 M Na_2_SO_4_ is the widest (1 V for 1 M Na_2_SO_4_, 0.4 V for 1 M H_2_SO_4_, and 0.4 V for 2 M NaOH). Since MBene-based and MoB-based materials are good electrocatalysts for hydrogen (HER) and oxygen evolution reactions (OER),^16,36^ HER and ORR easily occur in acid and alkaline electrolytes, limiting the voltage window largely. The areal capacitance results are summarized in Figure 5f. At low scan rates, the results showed that the performance order was 1 M H_2_SO_4_ > 1 M Na_2_SO_4_ > 2 M NaOH. This can be attributed to the cation migration rate, with H^+^ having a faster migration rate than Na^+^ due to its smaller size. However, long-duration electrochemical tests in acidic and alkaline electrolytes caused severe capacitance attenuation at high scan rates, making them unsuitable for energy storage.

**Figure 6:**
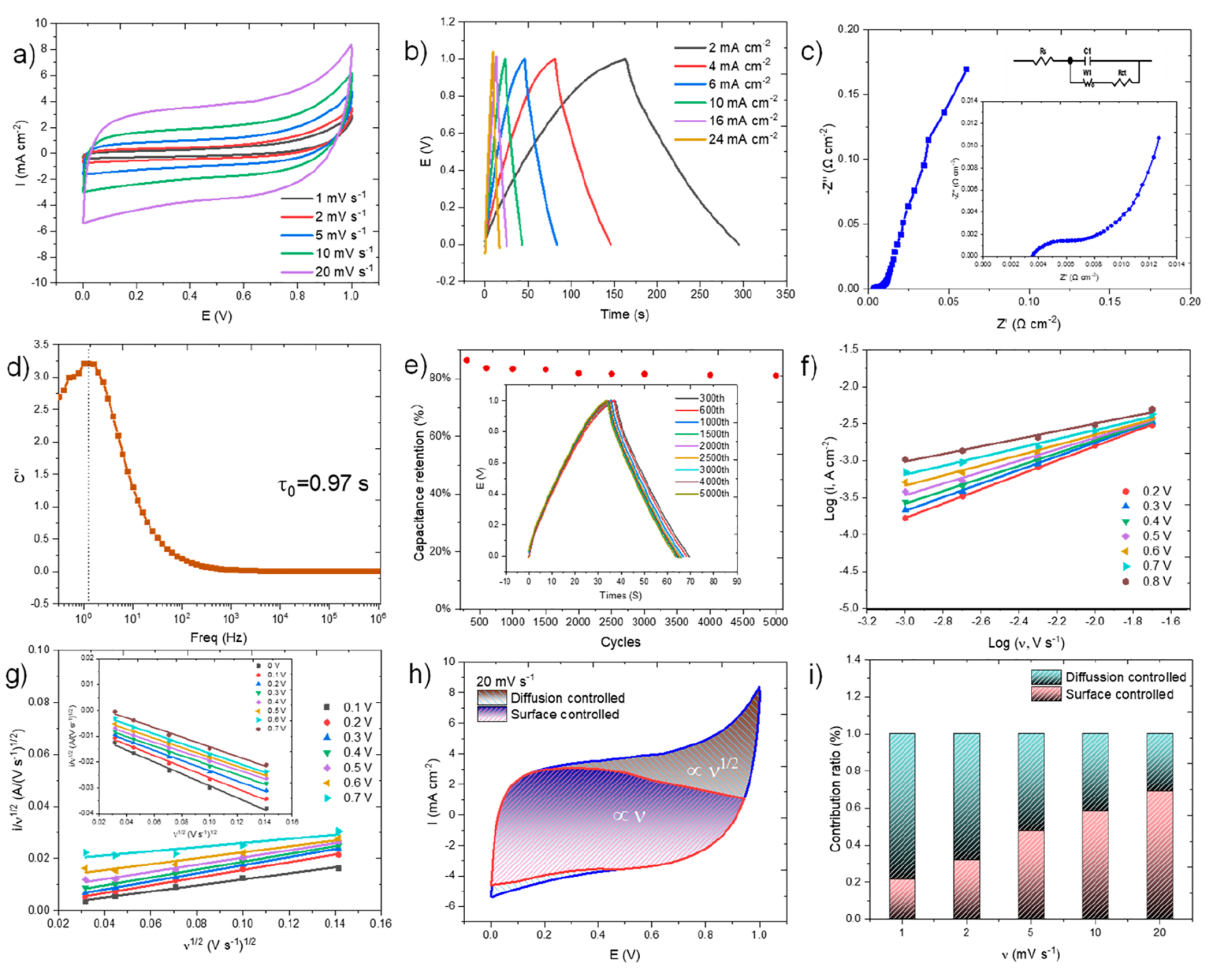
Electrochemical measurements for 1/24-MoAl_1–*x*_B in the 2-electrode setup (1 M Na_2_SO_4_ as electrolyte): (a) CV curves at different scan rates, (b) GCD curves at different charge–discharge speeds, (c) Nyquist plots obtained by EIS with enlargement and equivalent circuit in the inset, (d) evolution of imaginary part of the capacitance versus frequency, (e) cycling stability tested at 6 mA cm^–2^, (f) power-law dependence of current response versus scan rate at different voltages, (g) top current (inset is bottom current) response to the square root of scan rate versus the square root of the scan rate, (h) CV curves at the scan rate at 20 mV s^–1^ with the enclosed red area and a blue area representing for surface-contribution and diffusion-contribution, respectively, and (i) the proportions of the surface-controlled and diffusion-controlled capacitive contribution at different scan rates.

Further study has been conducted for the supercapacitor performance and energy storage mechanism of 1/24-MoAl_1–*x*_B in a 2-electrode configuration. Two 1/24-MoAl_1–*x*_B film electrodes sandwiched with an NKK separator were fabricated in a symmetric two-electrode configuration and tested in 1 M Na_2_SO_4_. The charge storage capacity of the electrode was primarily evaluated by CV measurement at different scan rates in a potential window of 0–1V (Figure 6a). The quasirectangular CV curve reflects the good conductivity of the film electrode. Table 2 summarizes the excellent performance of 1/24-MoAl_1–*x*_B and compares it to other MXene/molybdenum/boron-based electrodes (Table S6), with an areal capacitance of 2006.60 mF cm^–2^ (1 mV s^–1^) calculated from CV curves. Despite its higher loading mass (30 mg cm^–2^) due to the much higher molecular weight of MoAlB, the film electrode has a thickness of only 120 μm, which meets the thickness limit of thin film electrodes^37^ and achieves an outstanding volumetric capacitance of 166.67 F cm^–3^ for a single electrode at the scan rate of 1 mV s^–1^. The specific mass capacitance of 66.89 F g^–1^ here is considered moderate when compared to some low molecular weight active materials, such as active carbon^38,39^ and MXene/molybdenum/boron-based electrodes (Table S6), but the excellent areal capacitance suggests promising applications of MBenes in supercapacitors. Moreover, the specific mass capacitance could be significantly enhanced by further removing more Al and exposing more surface, demonstrating the potential for future improvements.

**Table 2:** Capacitance of the 1/24-MoAl_1–*x*_B Electrode Calculated from CV Curves

*v* (mV s^–1^)	*C*_*S*(device)_ (mF cm^–2^)	*C*_*S*(single-electrode)_ (mF cm^–2^)	*C*_*V*(single-electrode)_ (F cm^–3^)
1	1003.30	2006.60	167.21
2	699.63	1399.25	116.60
5	498.79	997.57	83.13
10	420.80	841.60	70.13
20	368.22	736.44	61.37

GCD curves (Figure 6b) with no obvious voltage drop at high charge–discharge speed and the almost symmetric shape show their perfect cycle life. It even has relatively high capacitance performances of 590.17 and 397.92 mF cm^–2^ at a charge–discharge rate of 2 mA cm^–2^ and a high charge–discharge rate of 24 mA cm^–2^, respectively (Table S7). The high performance could be attributed to its good ion diffusion and ultralow resistance as shown in Figure 6c: the intercept on the horizontal axis and the semicircular arc at the high frequency is related to the solution resistance for the formation of electrode–electrolyte interaction (*R*_S_) and the charge transfer resistance (*R*_ct_), respectively, which are calculated to be equal to 0.0037 and 0.0023 Ω cm^–2^ based on the equivalent circuit fitting shown in the inset of Figure 6c. These results are much lower than those of MXene-based electrodes (3.0 and 6.5 Ω cm^–2^ for bimetallic-sulfide@layered Ti_3_C_2_Tx-MXene,^40^ 1.109 and 0.0075 Ω cm^–2^ for Ti_3_C_2_Tx@Al,^26^ and 0.392 and 0.164 Ω cm^–2^ for PANI/small-sized MXene^41^). The complex model of capacitance was utilized to further confirm the impedance behavior (eqs S13 and S14).^42,43^ The relaxation time constant τ_0_ can be calculated according to the peak position for imaginary capacitance *C*″(ω) from the equation τ_0_ = 1/*f*_0_, where *f*_0_ is the frequency. A noticeably short time constant of τ_0_ = 0.97 s (Figure 6d) indicates its high charge transport kinetics. The cycling stability was tested by GCD at a charge–discharge speed of 6 mA cm^–2^. As shown in Figure 6e, the film electrode possesses excellent cycling durability and remains at 80.2% capacitance after 5000 cycles.

Generally, capacitance can be divided into surface-controlled capacitance and diffusion-controlled pseudocapacitance.^40,43^ Electrochemical kinetics analysis has been preliminarily analyzed by the CV method using eqs S15 and S16.^43,44^ The *b* value is determined from the plot of log(*i*) versus log(υ). For an ideal surface capacitance process, the *b* value is 1. The *b* value is 0.5 when only the pseudocapacitance process is observed. The *b* value is between 0.5 and 1 when both the capacitance processes coexist. As shown in Figure 6f, *b* values are in the range of 0.6–1, indicating that the surface-controlled capacitance and diffusion-controlled capacitance coexist during charge–discharge. Furthermore, the surface-controlled contribution to the overall current response was further quantified by conducting Dunn’s method (eqs S17 and S18),^35,43,44^ where *k*_1_*v* represents the surface-controlled contribution and *k*_2_*v*^1/2^ represents the diffusion-controlled contribution. The top current (inset is bottom current) responds to the square root of the scan rate versus the square root of the scan rate shown in Figure 6g. Even at a high scan rate of 20 mV s^–1^, the diffusion-controlled area still accounts for 30.78%, and the surface-controlled capacitive accounts for 69.22%, illustrated in Figure 6h. It has been found that with the increase in scan rate, the surface-controlled component increases from 21.54% to 69.22%, and the diffusion-controlled contribution decreases from 78.46% to 30.78% (Figures 6i and S3). Large interlayer structure contributes to the surface capacitance, and the high diffusion-controlled pseudocapacitance benefits from its ultrahigh conductivity. The hybrid charge storage mechanism is beneficial for increasing energy and high-power densities with excellent stability.

**Figure 7:**
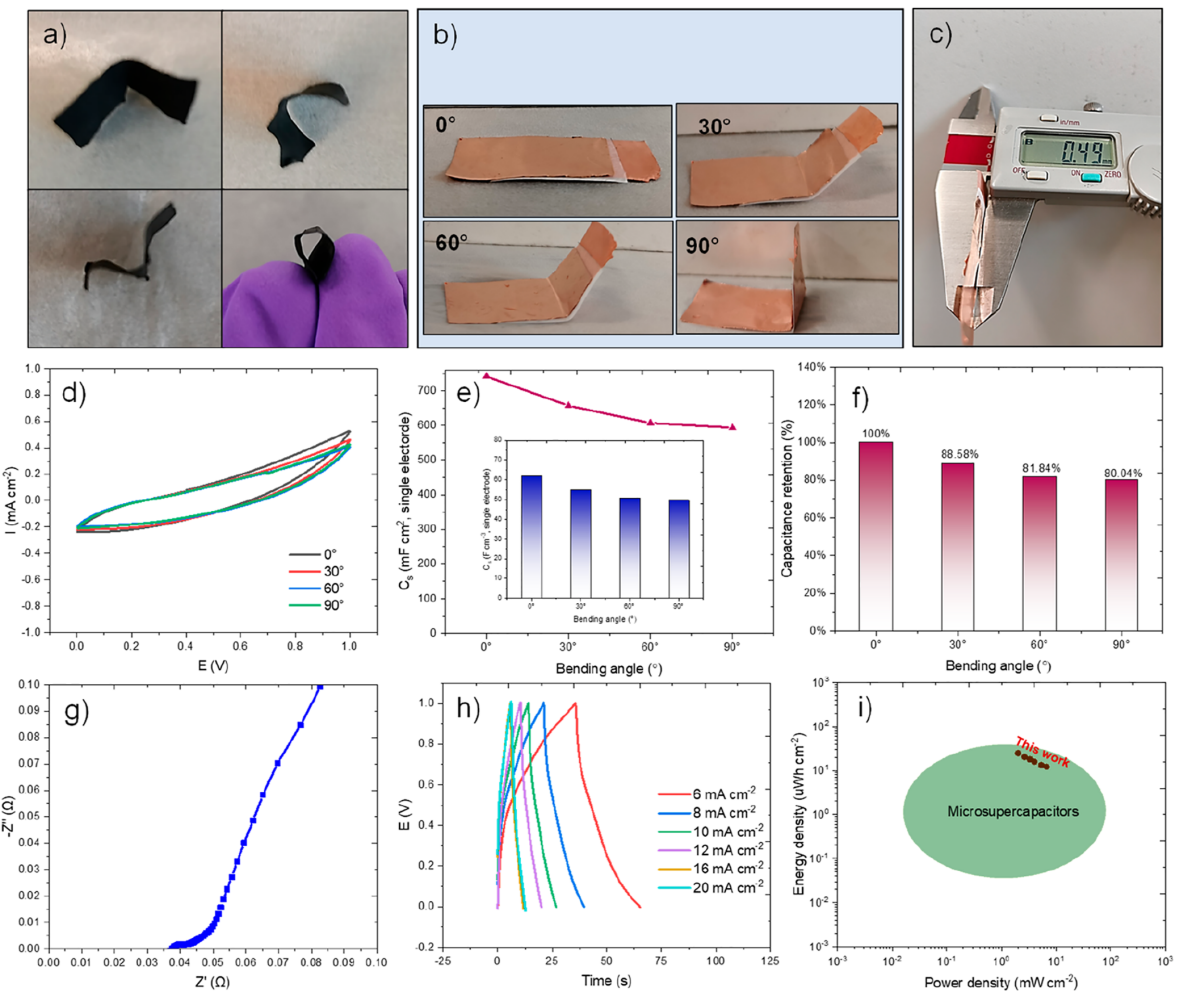
Fabrication and electrochemical measurements for 1/24-MoAl_1–*x*_B ASSS: (a) photographs of a 1 × 1.5 cm^2^ film electrode bent to different shapes, (b) photographs of ASSS bent to different bending angles (0°, 30°, 45°, and 90°), (c) thickness of the whole fabricated ASSS, (d) CV curves under different bending angles (0°, 30°, 45°, and 90°), (e) capacitance and (f) capacitance retention of the single electrode under different bending angles, (g) Nyquist plots obtained by EIS, (h) GCD curves, and (i) Ragone plots of this work resulting in microsupercapacitors area.^45^

A very flexible film electrode with as low as ∼120 μm thickness has been fabricated, which could be bent to different shapes, as shown in Figure 7a. A couple of flexible 1/24-MoAl_1–*x*_B film (1 × 1.5 cm^2^) electrodes were assembled into an ASSS by sandwiching an NKK separator with LiCl–PVA as the solid-state electrolyte and Cu foils as current collectors. The ASSS retains stability at different bending states, as shown in Figure 7b. The thickness of the fabricated ASSS was 490 μm after assembly to ASSS (Figure 7c). Figure 7d,e shows that the capacitance of a single electrode at 1 mV cm^–2^ is 741.6 mF cm^–2^ or 61.8 F cm^–3^ (370.8 mF cm^–2^ for the ASSS device), which exceeds many MXenes or other active material previously reported in symmetric ASSS (Table S8). When the device is bent from 0° to 30°, 45°, and 90°, the CV curves show negligible change, and the capacitances almost remained at 81% when bending to a high angle of 90° (Figure 7f), which is due to the low interfacial resistance (0.037 Ω) as in Figure 7g. It is possible to evaluate the energy power storage properties through GCD measurements shown in Figure 7h,i and Table S9. The device exhibits a good areal energy density of 24.65 μWh cm^–2^ at a power density of 2 mW cm^–2^ and maintains a value of 12.2 μWh cm^–2^ at a high-power density of 6.7 mW cm^–2^. These values are considered to be higher than those of the most previously reported microsupercapacitors since it lies in the upper right corner area of the Ragone plot (Figure 7i).^45^ This suggests that the 1/24-MoAl_1–*x*_B ASSS meets the requirements of powering some wearable devices and portable or on-chip electronics.^46^

## Conclusions

This research presents a novel approach for synthesizing multilayered MBenes MoAl_1–*x*_B using a mild, fluorine-free strategy of dilute alkali etching MoAlB to achieve different Al deintercalation rates. The etching route and chemical reaction were studied, and 1/24-MoAl_1–*x*_B with −OH terminal groups exhibited approximately 25% removal of Al at room temperature for 24 h using a 1 wt % NaOH solution, surpassing traditional etching solutions. The properties and energy storage mechanism of MBenes in supercapacitors were investigated for the first time. The capacitance of MoAl_1–*x*_B increased with an increasing Al removal rate and was better than those of traditional fluoride etching products. The 1/24-MoAl_1–*x*_B film electrode exhibited ultrahigh conductivity, superior cycle performance, and high areal capacitance. Furthermore, an all-solid-state supercapacitor device based on the flexible 1/24-MoAl_1–*x*_B film electrode delivered significant capacitance, high energy density, and power density even at a 90° bending angle. Our research findings indicate that two-dimensional transition metal borides (MBenes) have significant potential for use in supercapacitors and that their properties could be further improved by increasing the Al deintercalation rate, thus advancing their practical applications in energy storage.

## Supporting Information Available

Calculation methods and corresponding equations, more details of XPS analysis for MoAlB and 1/24-MoAl_1–*x*_B, SEM images of MoAlB in different resolutions, EDS spectrum of the dark phase in LiF/HCl-MoAl_1–*x*_B, the areal capacitance of MoAlB, 1/24-MoAl_1–*x*_B, and LiF/HCl-MoAl_1–*x*_B in 3 electrodes at different scan rates, comparison of the capacitance for MXene/molybdenum/boron-based electrodes, CV curves of 1/24-MoAl_1–*x*_B with separation between total current and surface-controlled capacitance at different scan rates, comparison of *C*_S_ for different ASSSs, energy density and power density of 1/24-MoAl_1–*x*_B ASSS (PDF)
