# Supplementary material for: Exploring the Potential of MBenes Supercapacitors: Fluorine-Free Synthesized MoAl1–xB with Ultrahigh Conductivity and Open Space
Source: ACS Appl Mater Interfaces. 2023 Jul 5;15(28):33560–70. doi: 10.1021/acsami.3c04301 (PMC10360035; doi:10.1021/acsami.3c04301)
Supplement: Supplementary file 1 — am3c04301_si_001.pdf [file am3c04301_si_001.pdf]

Exploring the Potential of MBenes Supercapacitors: Fluorine-Free Synthesized $MoAl_{1-x}B$ with Ultrahigh Conductivity and Open Space

Shudan Wei, Xiaojun Lai, Girish M Kale*

School of Chemical and Process Engineering, University of Leeds, LS2 9JT, Leeds, United Kingdom

Corresponding E-mail: G.M.Kale@leeds.ac.uk

1. Calculation Methods

1.1 Capacitance

In a 3-electrode set, the areal capacitance for the film electrode could be calculated by **Equation S1, S2:**[1]

$$C_{S(GCD)} = \frac{I\Delta t}{A\Delta V} \quad \text{(S1)}$$

$$C_{S(CV)} = \frac{\int i(V)dV}{A\Delta V v} \quad \text{(S2)}$$

In 2-electrode setup or all-solid-state supercapacitor (ASSS), the properties of film electrodes could be calculated by following **Equation S3~S8:**[2, 3]

$$C_{S(GCD,device)} = \frac{I\Delta t}{A\Delta V} \quad \text{(S3)}$$

$$C_{S(CV,device)} = \frac{\int i(V)dV}{A\Delta V v} \quad \text{(S4)}$$

$$C_{S(GCD,single-electrode)} = \frac{2I\Delta t}{A\Delta V} \quad \text{(S5)}$$

$$C_{S(CV,single-electrode)} = \frac{2\int i(V)dV}{A\Delta V v}$$

(S6)

$$C_{V(GCD,single-electrode)} = \frac{C_{S(GCD,single-electrode)}}{T} \quad \text{(S7)}$$

$$C_{V(CV,single-electrode)} = \frac{C_{S(CV,single-electrode)}}{T} \quad \text{(S8)}$$

where C_S (mF cm^{-2}), and C_V (mF cm^{-3}) are the areal capacitance and volumetric capacitance, respectively. GCD/CV means calculating from GCD or CV curves and single-electrode/device represents the capacitance for single electrode or the entire device. I, A, Δt, ΔV, and T are the applied current (mA), the area of the electrodes (cm^2), discharging time (s), the potential window (V), and the thickness of the film electrode (cm), respectively $\int i(V)dV$ is the integrated area of the enclosed CV curve, and v is CV scan rate (V s^{-1}).

1.2 Energy density and power density

Use **Equation S9~10** to calculate the energy density E and power density P for ASSS:[1, 4]

$$E = \frac{CV^2}{2} \tag{S9}$$

$$P = \frac{E}{\Delta t} \tag{S10}$$

1.3. Diffusion coefficient

Based on the EIS results, the diffusion coefficient (D) can be analyzed by fitting the real part (Z_{re}) of the impedance with the square root of the radial frequency ($\omega^{-1/2}$) as in the following **Equation S11, S12:**[5]

$$Z_{re} = R + \sigma\omega^{-1/2} \tag{S11}$$

$$D = \frac{R^2T^2}{2S^2n^4F^4C^2\sigma^2} \tag{S12}$$

Where R is the gas constant, T is the absolute temperature, S is the surface area of the electrode, n is the number of electronic transfers, F is the Faraday constant, C is the H^+ ion concentration, and σ is the Warburg factor related to Z_{re}.

1.4 Complex model of capacitance

The complex model of capacitance was used to further confirm the impedance behavior. The real $C'(\omega)$ and imaginary $C''(\omega)$ capacitances can be obtained:[4, 6]

$$C'(\omega) = -Z''(\omega)/(\omega|Z(\omega)|^2) \tag{S13}$$

$$C''(\omega) = Z'(\omega)/(\omega|Z(\omega)|^2) \tag{S14}$$

Where $Z(\omega)$ is the complex impedance, $Z'(\omega)$ and $Z''(\omega)$ represent the real part of $Z(\omega)$ and the imaginary part of $Z(\omega)$, respectively, and ω is the angular frequency ($\omega=2\pi f$). the relaxation time constant can be calculated according to the peak position for $C''(\omega)$ from the equation $\tau_0=1/f_0$, where f_0 is the frequency.

1.5 Electrochemical kinetics analysis

1) CV method

Electrochemical kinetics analysis has been preliminarily analyzed by the CV method: [5] [4]

$$i = av^b \tag{S15}$$

$$log(i) = blog(v) + \log(a) \tag{S16}$$

Where i represents the current, v represents the sweep rate, and a as well as b are the adjustable coefficients. The b-value is determined from the slope of the plot of $log(i)$ versus $log(v)$.

2) Dunn's method

The surface-controlled contribution to the overall current response was further quantified by conducting Dunn's method. The current response is proportional to the scan rate for a surface-controlled process, while the current response is proportional to the square root of the scan rate for a diffusion-controlled process as follows:[4, 5]

$$i(v) = k_1 v + k_2 v^{1/2} \tag{S17}$$

$$i(v)/v^{1/2} = k_1 v^{1/2} + k_2 \tag{S18}$$

Where $i(v)$ is the current response under a fixed potential, $k_1 v$ represents the surface-controlled contribution, $k_2 v^{1/2}$ represents the diffusion-controlled contribution, and v is the scan rate, where k_1 and as k_2 are constants.

2. XPS Analysis for MoAlB and 1/24-$MoAl_{1-x}B$

2.1 MoAlB

The species belonging to the MoAlB compound were extracted from the high-resolution spectra fitting shown in Figure 4 (lower spectra) and Table S2-S5. The XPS spectra of the Mo 3d region were fitted with 3 components: MoAlB, Mo^{4+} and Mo^{6+}. The first component belongs to Mo in the MoAlB compound, [7] while the other 2 are surface oxides.[8] The high-resolution spectra of the Al 2p region were fitted by two components MoAlB and Al_2O_3, the first belongs to Al species in the MoAlB compound [7] and the second belongs to Al_2O_3 surface oxide.[9] While the high-resolution XPS spectra

of B 1s were fitted by 2 components MoAlB and B_2O_3, the first is assigned to B species in the MoAlB compound [7] and the second is assigned to B_2O_3 surface oxide.[10] As for the high-resolution spectra of O 1s, it was fitted by two components Al oxide [9] and Mo oxide [11].

2.2 1/24-MoAl$_{1-x}$B

The fitted XPS spectra for 1/24-MoAl$_{1-x}$B was shown in Figure 4 (upper spectra) and table S2-S5. The fitted peak at binding energy (BE) of 228.53 eV (231.58eV) is assigned to MoAl$_{1-x}$B and is shifted by 0.75 eV higher than that for the MoAlB. This shift is due to the removal of Al and the introduction of surface terminations -OH. Such shift has also been observed for the M (metal) element when selectively etching the A element in MAX phases, forming MXenes.[12] The fitted peaks at 230.94 (233.78), and 232.75 (235.91) eV correspond to oxide states of Mo, Mo^{4+}, and Mo^{6+}, respectively, most likely originating from surface oxidation.[8] The peak of Al 2p for 1/24-MoAl$_{1-x}$B is also shifted from 73.4 eV to 73.87 eV because of the increasing valence of remaining Al and B 1s is shifted by 1eV to higher value because of the introduction of electrodes withdraw group -OH. The fitted XPS spectra of the O 1s regions show the presence of OH.

3 Supporting Figures and Tables

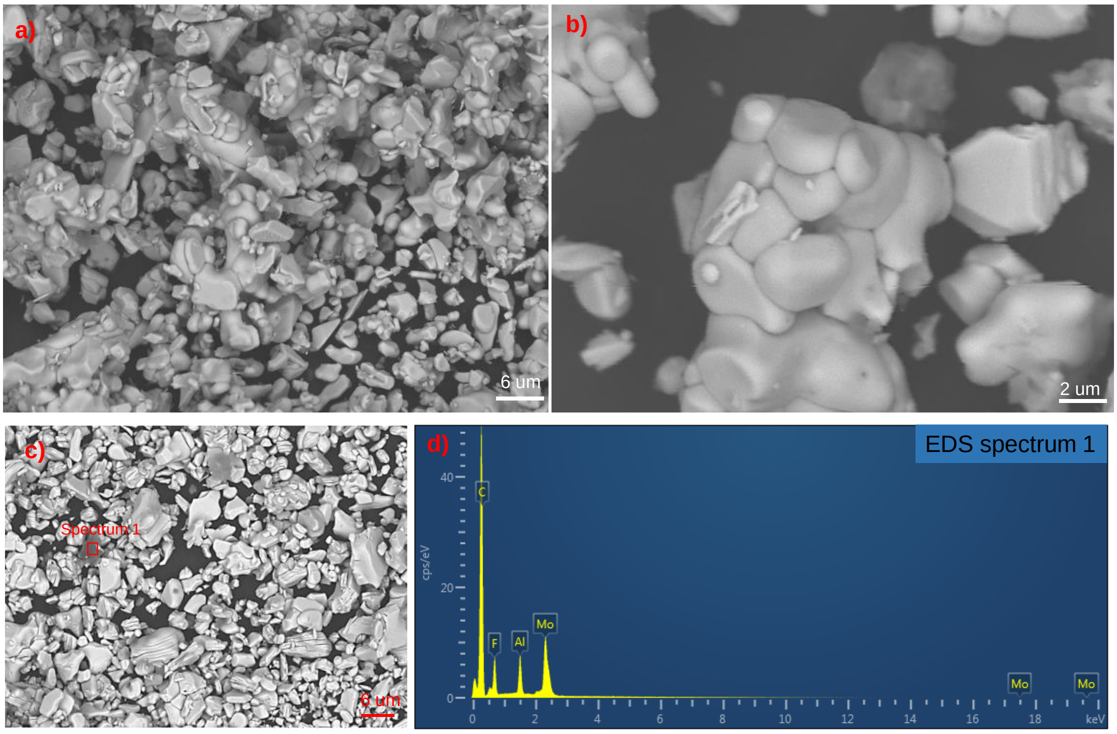

Figure S1 a), b) SEM images of MoAlB in different resolutions, c) SEM image of LiF/HCl-$MoAl_{1-x}B$, d) EDS spectrum of the dark phase in LiF/HCl-$MoAl_{1-x}B$.

Table S1 Atomic ratio of dark phase (spectrum 1) in LiF/HCl-$MoAl_{1-x}B$

**Element**	**Line Type**	**Atomic %**
F	K series	8.08
Al	K series	1.46
Mo	L series	1.77
C	K series	88.68
Total		100.00

Table S2. XPS peak fitting results for Mo 3d region

Samples	Assigned to	BE (eV)	Fraction	References
MoAlB	MoAlB	227.78 (231.10)	0.67	[7]
	Mo^{4+}	230.85 (234.20)	0.1	[8]
	Mo^{6+}	233.03 (236.08)	0.23	
1/24-$MoAl_{1-x}B$	$MoAl_{1-x}B$	228.53 (231.58)	0.11	This work
	Mo^{4+}	230.94 (233.78)	0.59	[8]
	Mo^{6+}	232.75 (235.91)	0.3	

Table S3 XPS peak fitting results for Al 2p region

Samples	Assigned to	BE (eV)	Fraction	References
MoAlB	MoAlB	73.4	0.24	[7]
	Al_2O_3	75.3	0.76	[9]
1/24-$MoAl_{1-x}B$	$MoAl_{1-x}B$	73.87	0.2	This work
	Al_2O_3	74.71	0.8	[9]

Table S4. XPS peak fitting results for B 1s region

Samples	Assigned to	BE (eV)	Fraction	References
MoAlB	MoAlB	188.91	0.45	[7]
	B_2O_3	192.85	0.55	[10]
1/24-$MoAl_{1-x}B$	$MoAl_{1-x}B$	189.97	0.17	This work
	B_2O_3	192.94	0.83	[10]

Table S5 XPS peak fitting results for O 1s region

Samples	Assigned to	BE (eV)	Fraction	References
MoAlB	Mo-O	529.91	0.46	[13]
	Al_2O_3	531.50	0.54	[9]
$1/24-MoAl_{1-x}B$	Mo-O	529.80	0.57	[13]
	Al_2O_3	531.41	0.13	[9]
	-OH	533.29	0.30	[13]

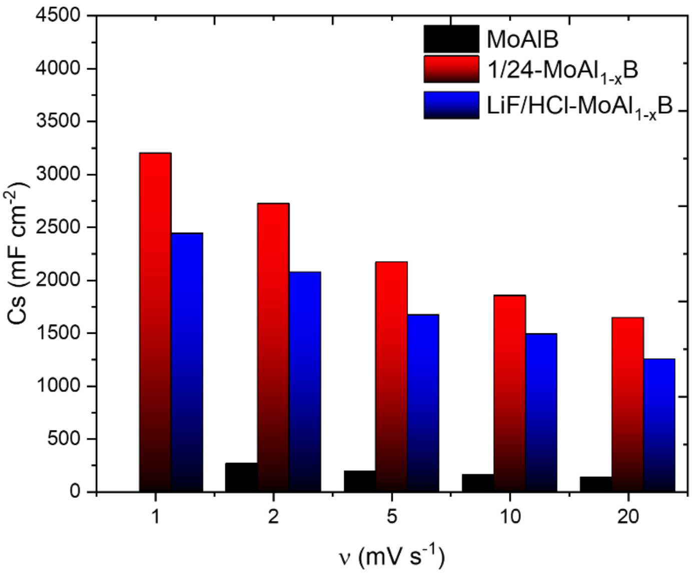

Figure S2 Areal capacitance of MoAlB, 1/24-$MoAl_{1-x}B$ and LiF/HCl-$MoAl_{1-x}B$ in 3-electrodes at different scan rates.

Table S6 Comparison of the capacitance for MXene/Molybdenum/Boron-based electrodes

Samples	Areal Capacitance ($mF\ cm^{-2}$)	Gravimetric capacitance ($F\ g^{-1}$)	Current density/ Scan rate	Electrolyte	Ref.
$Ti_3C_2T_X$@Al	1087	54.35	1 mA cm^{-2}	0.5 M Na_2SO_4	[2]
$Ti_3C_2T_X$ (T_X=SO_4)	1399	93.27	1 mV s^{-1}	0.5 M Na_2SO_4	[14]
$NiCo_2O_4$@TiN	679	/	2 mA cm^{-2}	1 M KOH	[15]
MXene-RuO_2	416	401	10 mV s^{-1}	1 M H_2SO_4	[16]
RGO/MoS_2/PEDOT	241.81	626.45	0.5 mA cm^{-2} /1 A g^{-1}	1 M H_2SO_4	[17]
MoN_x/TiN	121.50	174.83	0.3 mA cm^{-2} /1.5 A g^{-1}	1.0 M LiOH	[18]
Boron nanowire-carbon fiber cloth	60.2	78.88	0.2 mA cm^{-2} /2 A g^{-1}	4 M KOH	[19]
Oxygen defective boron nanosheet	141.55	14.06	2 A g^{-1} /20 mA cm^{-2}	1 M H_2SO_4	[20]
$MoAl_{1-x}B$	2006.60	66.89	1 mV s^{-1}	1 M Na_2SO_4	This work

Table S7. Cs ($mF\ cm^{-2}$) of 1/24-$MoAl_{1-x}B$ calculated from GCD results in 2-electrode setup

I ($mA\ cm^{-2}$)	Device (Cs, $mF\ cm^{-2}$)	Single electrode (Cs, $mF\ cm^{-2}$)
2	295.09	590.17
4	292.02	584.04
6	249.26	498.52
10	216.55	433.10
16	205.14	401.28
24	198.96	397.92

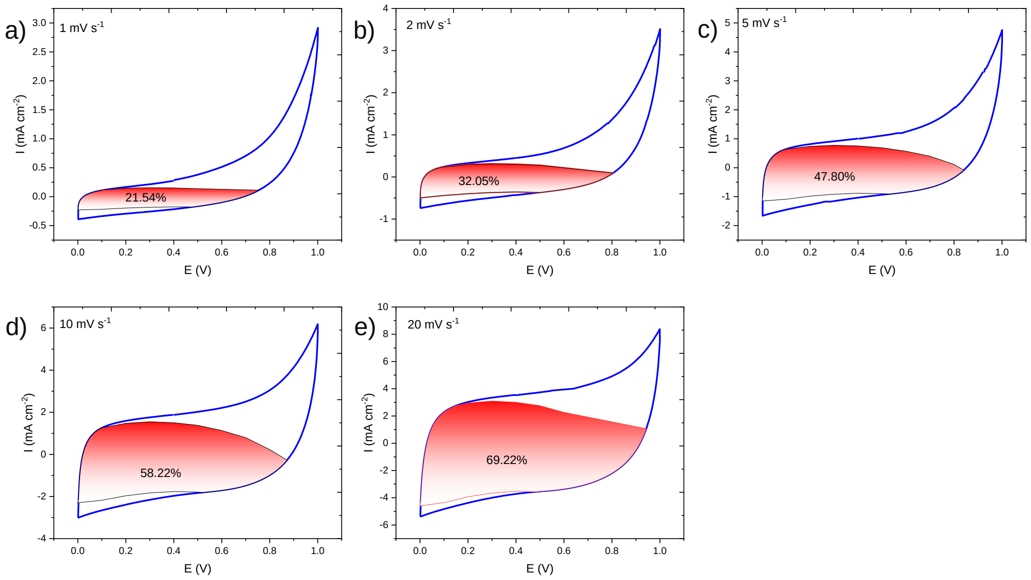

Figure S3 CV curves of 1/24-$MoAl_{1-x}B$ with separation between total current (blue line) and surface-controlled capacitance (red shadow) at a) 1, b) 2, c) 5, d) 10, and e) 20 mV s^{-1}.

Table S8 Summary and comparison of C_S for ASSSs

**Electrode**	**Electrolyte**	**Test condition**	**Cs (devices, mF cm^{-2})**	**Ref.**
$Ti_3C_2T_X$@Al	PVA-H_2SO_4	1 mV s^{-1}	242.3	[2]
$NiCo_2O_4$@TiN	PVA-KOH	0.3 mA cm^{-2}	35	[15]
Ti_3C_2Tx films	PVA-H_2SO_4	10 mV s-1	220	[21]
RGO/MoS_2/PEDOT	PVA-H_3PO_4	0.104 mA cm^{-2}	10.35	[17]
CT-Ti_3C_2@V_2O_5	PVA-LiCl	1 mV s^{-1}	477	[22]
Compact graphene/MoS_2 films	PVA-H_3PO_4	0.3 mA cm^{-2}	70	[23]
Boron nanowire-carbon fiber cloth	PVA-H_2SO_4	10 mV s^{-1}	17.5	[19]
Ag-doped PEDOT:PSS/CNT	PVA-H_3PO_4	0.1 mA cm^{-2}	64	[24]
SA-CNT films	PVA-H_3PO_4	1 A g^{-1}	3.8	[25]
$MoAl_{1-x}B$	PVA-LiCl	1 mV s^{-1}	370.8	This work

Table S9 Electrochemical properties of 1/24-$MoAl_{1-x}B$ calculated from GCD results in ASSS.

**I (mA cm^{-2})**	**Device Cs (mF cm^{-2})**	**Single electrode C_S (mF cm^{-2})**	**E (uWh cm^{-2})**	**P (mW cm^{-2})**
6	177.48	354.96	24.65	2
8	147.31	294.61	20.47	2.67
10	128.93	257.87	17.91	3.33
12	114.08	228.16	15.84	4
16	95.15	190.29	13.21	5.33
20	87.73	175.47	12.19	6.67

References:

1. Li, Q. Q.; Liu, M. J.; Huang, F. Z.; Zuo, X. Q.; Wei, X.; Li, S. K.; Zhang, H., Co_9S_8@MnO_2 core-shell defective heterostructure for High-Voltage flexible supercapacitor and Zn-ion hybrid supercapacitor. *Chem. Eng. J.* **2022,** *437*.
2. Guo, M.; Liu, C. B.; Zhang, Z. Z.; Zhou, J.; Tang, Y. H.; Luo, S. L., Flexible Ti_3C_2Tx@Al electrodes with Ultrahigh Areal Capacitance: In Situ Regulation of Interlayer Conductivity and Spacing. *Adv. Funct. Mater.* **2018,** *28* (37).
3. Amir, F. Z.; Pham, V. H.; Schultheis, E. M.; Dickerson, J. H., Flexible, all-solid-state, high-cell potential supercapacitors based on holey reduced graphene oxide/manganese dioxide nanosheets. *Electrochim. Acta* **2018,** *260*, 944-951.
4. Li, X. L.; Yuan, L. B.; Liu, R.; He, H. N.; Hao, J. N.; Lu, Y.; Wang, Y. M.; Liang, G. M.; Yuan, G. H.; Guo, Z. P., Engineering Textile Electrode and Bacterial Cellulose Nanofiber Reinforced Hydrogel Electrolyte to Enable High-Performance Flexible All-Solid-State Supercapacitors. *Adv. Energy Mater.* **2021,** *11* (12).
5. Ge, P.; Hou, H. S.; Li, S. J.; Yang, L.; Ji, X. B., Tailoring Rod-Like $FeSe_2$ Coated with Nitrogen-Doped Carbon for High-Performance Sodium Storage. *Adv. Funct. Mater.* **2018,** *28* (30).
6. Yan, J.; Ren, C. E.; Maleski, K.; Hatter, C. B.; Anasori, B.; Urbankowski, P.; Sarycheva, A.; Gogotsi, Y., Flexible MXene/Graphene Films for Ultrafast Supercapacitors with Outstanding Volumetric Capacitance. *Adv. Funct. Mater.* **2017,** *27* (30).
7. Natu, V.; Kota, S. S.; Barsoum, M. W., X-ray photoelectron spectroscopy of the MAB phases, MoAlB, M2AlB2 (M = Cr, Fe), Cr3AlB4 and their binary monoborides. *J.Euro. Ceram. Soc.* **2020,** *40* (2), 305-314.
8. Escamilla, R.; Carvajal, E.; Cruz-Irisson, M.; Morales, F.; Huerta, L.; Verdin, E., XPS study of the electronic density of states in the superconducting Mo2B and Mo2BC compounds. *J. Mater. Sci.* **2016,** *51* (13), 6411-6418.
9. Strohmeier, B. R. J. S. S. S., Zinc aluminate ($ZnAl_2$O4) by XPS. *Strohmeier* **1994,** *3* (2), 128-134.

10. Burke, A. R.; Brown, C. R.; Bowling, W. C.; Glaub, J. E.; Kapsch, D.; Love, C. M.; Whitaker, R. B.; Moddeman, W. E., Ignition mechanism of the titanium–boron pyrotechnic mixture. *Surf. Interface. Anal.* **1988,** *11* (6-7), 353-358.
11. Zhou, J.; Palisaitis, J.; Halim, J.; Dahlqvist, M.; Tao, Q. Z.; Persson, I.; Hultman, L.; Persson, P. O. A.; Rosen, J., Boridene: Two-dimensional $Mo_{4/3}B_{2-x}$ with ordered metal vacancies obtained by chemical exfoliation. *Science* **2021,** *373* (6556), 801-805.
12. Halim, J.; Cook, K. M.; Naguib, M.; Eklund, P.; Gogotsi, Y.; Rosen, J.; Barsoum, M. W., X-ray photoelectron spectroscopy of select multi-layered transition metal carbides (MXenes). *Appl. Surf. Sci.* **2016,** *362*, 406-417.
13. Ding, X.; Ho, W.; Shang, J.; Zhang, L., Self doping promoted photocatalytic removal of no under visible light with Bi_2MoO6: Indispensable role of superoxide ions. *Appl. Catal. B* **2016,** *182*, 316-325.
14. Guo, M.; Geng, W. C.; Liu, C. B.; Gu, J. Y.; Zhang, Z. Z.; Tang, Y. H., Ultrahigh Areal Capacitance of Flexible MXene Electrodes: Electrostatic and Steric Effects of Terminations. *Chem. Mater.* **2020,** *32* (19), 8257-8265.
15. Wang, R. Q.; Xia, C.; Wei, N. N.; Alshareef, H. N., NiCo2O4@TiN Core-shell Electrodes through Conformal Atomic Layer Deposition for All-solid-state Supercapacitors. *Electrochim. Acta* **2016,** *196*, 611-621.
16. Jiang, Q.; Kurra, N.; Alhabeb, M.; Gogotsi, Y.; Alshareef, H. N., All Pseudocapacitive MXene-RuO_2 Asymmetric Supercapacitors. *Adv. Energy Mater.* **2018,** *8* (13).
17. Chen, Y.; Bai, J. G.; Yang, D. Y.; Sun, P.; Li, X., Excellent performance of flexible supercapacitor based on the ternary composites of reduced graphene oxide/molybdenum disulfide/poly (3,4-ethylenedioxythiophene). *Electrochim. Acta* **2020,** *330.*
18. Xie, Y. B.; Tian, F., Capacitive performance of molybdenum nitride/titanium nitride nanotube array for supercapacitor. *Mater. Sci. Eng. B Solid State Mater. Adv. Technol.* **2017,** *215*, 64-70.

19. Xue, Q.; Gan, H. B.; Huang, Y.; Zhu, M. S.; Pei, Z. X.; Li, H. F.; Deng, S. Z.; Liu, F.; Zhi, C. Y., Boron Element Nanowires Electrode for Supercapacitors. *Adv. Energy Mater.* **2018,** *8* (20).
20. Joshi, A.; Tomar, A. K.; Singh, G.; Sharma, R. K., Engineering oxygen defects in the boron nanosheet for stabilizing complex bonding structure: An approach for high-performance supercapacitor. *Chem. Eng. J.* **2021,** *407*.
21. Yang, S.; Zhang, P. P.; Wang, F. X.; Ricciardulli, A. G.; Lohe, M. R.; Blom, P. W. M.; Feng, X. L., Fluoride-Free Synthesis of Two-Dimensional Titanium Carbide (MXene) Using A Binary Aqueous System. *Angew. Chem. Int. Ed.* **2018,** *57* (47), 15491-15495.
22. Zhang, Z. Z.; Guo, M.; Tang, Y. H.; Liu, C. B.; Zhou, J.; Yuan, J. L.; Gu, J. Y., High areal capacitance of vanadium oxides intercalated Ti3C2 MXene for flexible supercapacitors with high mass loading. *Nanotechnol.* **2020,** *31* (16).
23. Li, N.; Lv, T.; Yao, Y.; Li, H. L.; Liu, K.; Chen, T., Compact graphene/MoS2 composite films for highly flexible and stretchable all-solid-state supercapacitors. *J. Mater. Chem. A* **2017,** *5* (7), 3267-3273.
24. Pang, H.; Li, X. R.; Zhao, Q. X.; Xue, H. G.; Lai, W. Y.; Hu, Z.; Huang, W., One-pot synthesis of heterogeneous Co3O4-nanocube/Co(OH)(2)-nanosheet hybrids for high-performance flexible asymmetric all-solid-state supercapacitors. *Nano Energy* **2017,** *35*, 138-145.
25. Song, L.; Cao, X. B.; Li, L.; Wang, Q. D.; Ye, H. T.; Gu, L.; Mao, C. J.; Song, J. M.; Zhang, S. Y.; Niu, H. L., General Method for Large-Area Films of Carbon Nanomaterials and Application of a Self-Assembled Carbon Nanotube Film as a High-Performance Electrode Material for an All-Solid-State Supercapacitor. *Adv. Funct. Mater.* **2017,** *27* (21).
